# Effect- and Performance-Based Auditory Feedback on Interpersonal Coordination

**DOI:** 10.3389/fpsyg.2018.00404

**Published:** 2018-03-29

**Authors:** Tong-Hun Hwang, Gerd Schmitz, Kevin Klemmt, Lukas Brinkop, Shashank Ghai, Mircea Stoica, Alexander Maye, Holger Blume, Alfred O. Effenberg

**Affiliations:** ^1^Institute of Sports Science, Leibniz University Hannover, Hannover, Germany; ^2^Institute of Microelectronic Systems, Leibniz University Hannover, Hannover, Germany; ^3^Department of Neurophysiology and Pathophysiology, University of Hamburg, Hamburg, Germany

**Keywords:** auditory feedback, collaborative task, interpersonal coordination, movement sonification, sensorimotor contingencies theory

## Abstract

When two individuals interact in a collaborative task, such as carrying a sofa or a table, usually spatiotemporal coordination of individual motor behavior will emerge. In many cases, interpersonal coordination can arise independently of verbal communication, based on the observation of the partners' movements and/or the object's movements. In this study, we investigate how social coupling between two individuals can emerge in a collaborative task under different modes of perceptual information. A visual reference condition was compared with three different conditions with new types of additional auditory feedback provided in real time: effect-based auditory feedback, performance-based auditory feedback, and combined effect/performance-based auditory feedback. We have developed a new paradigm in which the actions of both participants continuously result in a seamlessly merged effect on an object simulated by a tablet computer application. Here, participants should temporally synchronize their movements with a 90° phase difference and precisely adjust the finger dynamics in order to keep the object (a ball) accurately rotating on a given circular trajectory on the tablet. Results demonstrate that interpersonal coordination in a joint task can be altered by different kinds of additional auditory information in various ways.

## Introduction

Researchers have recently focused on different modes of non-verbal communication concerning interpersonal coordination (e.g., mimicry, gestures, and facial expressions) as a basis of social interaction (Vicaria and Dickens, 2016). These kinds of nonverbal behavior can cause spatiotemporal coordination and support affective entrainment between two or more individuals (Phillips-Silver and Keller, 2012). Although it can be helpful to verbally share action plans and strategies, verbal communication might be too slow when one needs to instantly react to others' actions on a joint task (Knoblich and Jordan, 2003). Even in basic communication, concerning mother-child-dyads, it is important that two individuals immediately mediate information to drive entrainment (Phillips-Silver and Keller, 2012). Nonverbal communication can be realized via a broad spectrum of perceptual modalities, like visual, kinesthetic, tactile, or auditory systems, to support emergent coordination (Marsh et al., 2009). For example, Waterhouse et al. (2014) reported that two dancers nonverbally coordinated during their choreography performance. They synchronized the same movements or aligned the onset of different movements, relying on visual cues from their body movement as well as on auditory cues from breath and stepping sounds.

Commonly, if the amount of information is enhanced within a certain perceptual modality, interpersonal coordination will benefit from temporal synchronization (Knoblich and Jordan, 2003; Schmidt and Richardson, 2008). This is also given for the auditory domain: Musicians, performing in a joint action setting (e.g., orchestra, musical ensemble), regularly monitor auditory performance of their own, their co-performers' and the joint action outcomes to allow a smooth performance (Loehr et al., 2013). Likewise, Goebl and Palmer (2009) reported that auditory and visual information might function in a complementary fashion to support each other: During a joint action task, pianists produced exaggerated cues for their co-performers by finger movements when the auditory feedback was reduced or removed, which is possibly a compensatory mechanism in the visual domain to align co-performers actions (Repp and Keller, 2004). The important role of the auditory feedback for managing temporal synchrony during interpersonal coordination has been reported repeatedly (Goebl and Palmer, 2009; Demos et al., 2017; Vicary et al., 2017). Demos et al. (2017) compared asynchrony in the tone onset of expert pianists during a recorded and joint performance. The authors reported increased asynchronies once the auditory feedback was removed during the duet performance, confirming strong effects of auditory feedback on temporal synchronization in a joint task.

Demos et al. (2012) compared spontaneous interpersonal coordination under different combinations of auditory and visual information during a rhythmical rocking chair task. The authors reported that instantaneous coordination was enhanced with audio information alone (moving-chair sound, non-task-related music), compared to the condition with neither audio nor vision. In the audio-visual condition, the authors showed that the benefits of moving-chair attendant sound were much higher than in all other conditions, indicating enhanced spontaneous coordination compared to both vision-only and audio-only conditions (see also Schmidt and Richardson, 2008). However, Demos et al. (2012) observed less interpersonal coordination with non-task-related music compared to the moving-chair attendant sound condition, and even to the vision alone condition. Authors indicated that audio-visual feedback does not always lead to a positive effect, but it can cause interference. In an experiment on predictions of opponent's fencing attacks, Allerdissen et al. (2017) also reported that novices showed less performance in the audio-visual condition than in the visual-only condition. Allerdissen et al. (2017) explained that the meaningless additional auditory information might induce cognitive overload. Demos et al. (2012) reasoned that the spontaneous coordination would result from emergent perceptuo-motor couplings in the brain (Kelso, 1995). This can induce co-activation between auditory and motor cortices, so that additional auditory information can enhance synchronization (Bangert et al., 2006; Schmitz and Effenberg, 2017).

Research on additional auditory information related to motion has been reported recently. Vesper et al. (2013), for instance, asked a pair of participants to perform forward jumps next to each other, providing auditory and visual information about the partner's landing positions. Authors showed that the information aided participants to coordinate with each other, supporting both inter- and intra-personal coordination. In a study on audio-based perception of movements, Murgia et al. (2012) showed that participants are able to identify their own golf swing sounds. This study highlights the importance of temporal factors on self-other-discrimination because participants wrongly recognized golf swing sounds from others as their own sounds when the relative timing and the overall duration of movements are similar. On the other hand, a study from Kennel et al. (2014) found no effect of movement rhythm on self-other-discrimination in hurdling performance. The authors concluded that self-other discrimination of movement sounds is achieved by the individuality of sounds that activates one's own sensorimotor memory. They also argued that the larger number of appropriate internal models (e.g., sensorimotor, visual, auditory) enable participants to more accurately reproduce their movements. Furthermore, Keller (2012) suggested that online perceptual information might enhance the anticipation of one's own action as well as the co-performer's action in terms of developing common predictive internal models (Keller and Appel, 2010; Keller, 2012). From a neurophysiological aspect, it was suggested that auditory information possibly allows phase correction through a neural pathway across subsections of the cerebellum, which are connected to motor and auditory cortices (Keller et al., 2014). Periodic correction is, furthermore, enhanced with auditory feedback by additional recruitment of a corticothalamic network which includes the basal ganglia, prefrontal cortex, medial frontal cortex, and parietal cortex (Repp and Su, 2013; Keller et al., 2014).

Furthermore, recent studies have demonstrated the beneficial effects of real-time kinematic auditory feedback for enhancing motor control and learning (Effenberg, 2005; Effenberg et al., 2016). Even though it was in an individual setting, Effenberg et al. (2016) suggested that additional real-time auditory feedback enhances motor learning precisely in terms of a steeper temporal course for the development of motor representations. When mapped onto the kinematic and dynamic movement patterns, the additional real-time movement information might enhance the development of sensorimotor representation below the level of consciousness (Effenberg, 2005; Effenberg et al., 2016). This auditory feedback can be implemented in terms of both effect-based auditory feedback (EAF) and performance-based auditory feedback (PAF). Additional performance-based information provides feedback related to the quality of movement, whereas the effect-based information relays feedback of the result (Magill and Anderson, 2007; Schmidt and Wrisberg, 2008). Both the “knowledge of performance” (KP) and the “knowledge of result” (KR) are important for motor learning (Schmidt and Wrisberg, 2008). Several studies have reported the benefits of performance-based information on learning (Weeks and Kordus, 1998; Nunes et al., 2014; Sharma et al., 2016). Nevertheless, in situations when the feedback of performance is reduced, the impact of effect-based information is usually increased (Winstein, 1991; Schmidt and Wrisberg, 2008; Sharma et al., 2016). These types of feedback have been compared in the context of motor learning. We apply both types of feedback to the cooperative task in our study in order to explore their impact on interpersonal coordination.

**Figure 1:**
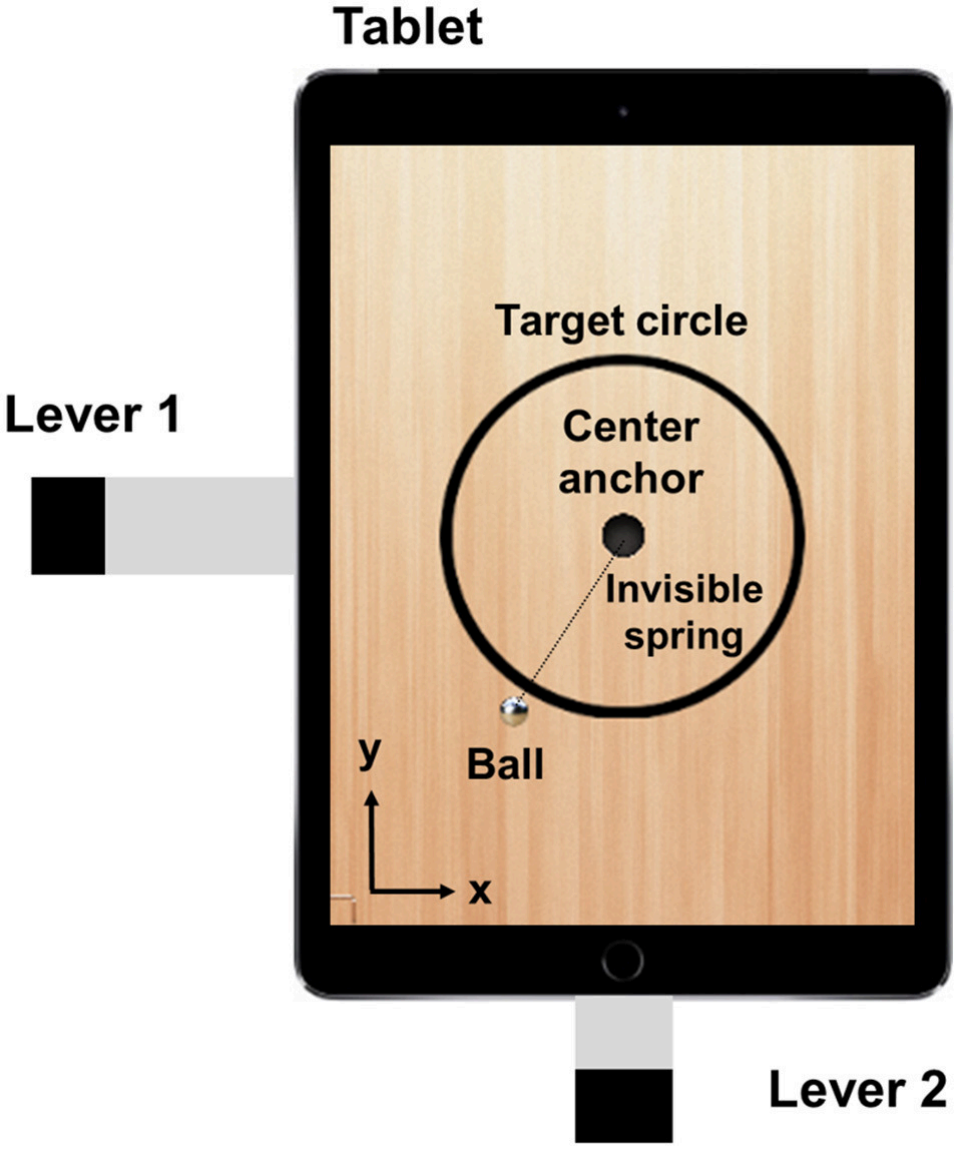
Top view of the tablet screen and levers fixed to the casing.

In this study, we developed a novel paradigm which we call the *tetherball paradigm*. The paradigm was implemented on a tablet computer (hereinafter called “tablet”) as shown in Figure 1. With rhythmical tilt-movements, a pair of participants had to accelerate a bound metal ball to revolve around the center of the scene (Figure 1). This task allows the analysis of joint performance by measuring the spatial error between the ball trajectory (controlled by both participants) and the circular target trajectory. Apart from visual information about the performance of both co-actors (the realized tilt in their own and their co-actor's axis) and about its effect (the deviation of the revolving ball from the target trajectory), we added different kinds of acoustic information to the paradigm. The feedback types correspond to the information about the performance. Although the action effect that is usually only available in the visual domain, PAF was generated from the tilt of the axes of the tablet and EAF was generated from the trajectory of the ball. The auditory information was based on the same features as the visual information (performance: tablet tilt; effect: ball trajectory). It may, nevertheless, affect the participants' perception in a different way because the auditory system is especially powerful in the temporal analysis of acoustic events, as well as in pace and rhythm specification and discrimination (Collier and Logan, 2000; Murgia et al., 2017). Furthermore, it is highly effective not only in the assessment of smoothness and regularity, but also in the synchronization and phase couplings and the adjustments of actions to external events (Repp and Penel, 2002). Therefore, we expect a better task performance, a stronger interpersonal coordination and a higher level of collaboration experience due to the additional involvement of the auditory perceptual system.

We compared three different audio-visual conditions [EAF, PAF, combined EAF and PAF (CAF)] to a visual condition (VF; no audio). For the PAF condition, we used a rhythmical sound which is in line with a recent research by Demos et al. (2012). EAF is a melodic sound (non-rhythmical sound) of integrated dynamics, which is created when two agents' joint actions result in a rotation of the ball. We intended to avoid a rhythmical feature in EAF because this might have allowed participants to identify the effect of their own movement effect within the effect sound. We decided to positively hypothesize according to previous literature (Vesper et al., 2013; Effenberg et al., 2016). In each condition, we evaluated the reduction of the trajectory error as a measure of task performance with on-going training as well as the cross-correlation of two participants' actions as a measure of their temporal synchronization. Participants were also asked to report their subjective experience of the coordination. With respect to these data, the following hypotheses were tested:

H1: Faster error reduction in the task is achieved when participants are provided with additional (a) effect-based, (b) performance-based, and (c) both combined auditory feedback.

H2: Cross-correlation in the participants' actions is stronger when participants are provided with (a) effect-based, (b) performance-based, and (c) both combined auditory feedback.

H3: Subjective ratings of the sense of interpersonal coordination are more positive when participants are additionally provided with (a) effect-based, (b) performance-based, and (c) both combined auditory feedback.

## Materials and methods

### Participants

We tested 72 healthy participants (30 females and 42 males; 24.8 ± 3.3 years) for normal eyesight and hearing abilities. Thirty-six pairs of participants were divided into four groups, corresponding to the four different conditions, so that each group consisted of nine pairs. Participants were randomly assigned to couples and the only criterion was “same-sex pair.” We also instructed them to use the dominant hand. The study was ethically approved by the Ethics Committee of Leibniz University Hannover.

### System specifications

The paradigm was implemented in Objective-C for iOS 10.2 on an iPad Air (Apple Inc.). Screen resolution was 1,024 × 768 at 60 Hz refresh rate. Accelerometers in the iPad were also sampled at 60 Hz. We used the Csound 6.0 (open-source code under LGPL) and Chipmunk2D Pro (Howling Moon Software) for the auditory feedback and physical implementation, respectively. The participants wore the headphones, Beyerdynamic DT-100. The audio signal was divided by a 4-channel stereo headphone amplifier, Behringer MicroAMP HA400.

### Design and stimuli

**Figure 2:**
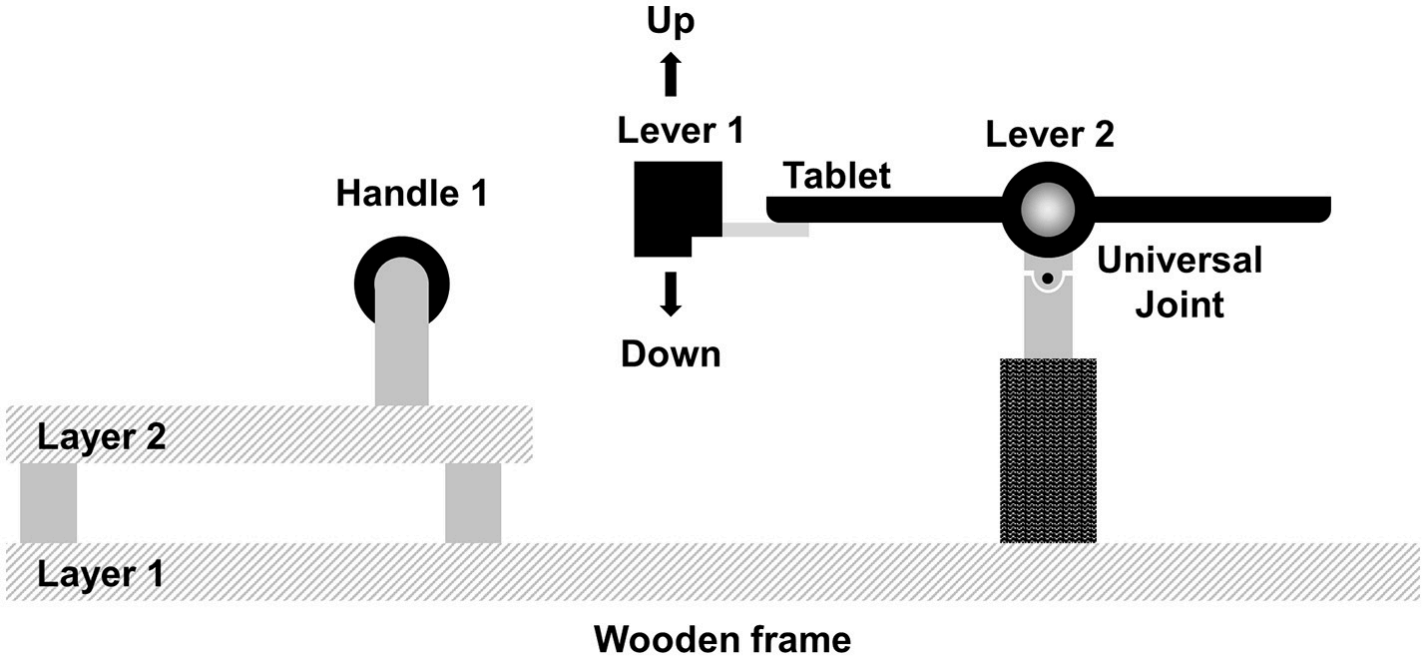
Side view of the apparatus which supports the tablet and allows mechanical movement of the tablet.

Figure 1 shows the main screen of the tablet application. The main components are the ball that is connected to the center by an invisible spring, the circular target trajectory continuously displayed on the tablet screen, and the levers fixed on both sides of the tablet. The tablet displays the components at XGA resolution, in which the ball radius is 30 pixels (px) and the radius of the target circle is 232.5 px (thickness: 15 px). The ball position refers to the center of the ball, expressed in x-y Cartesian coordinates. The ball is connected to the center anchor with an invisible elastic spring. The spring force is strong enough to pull the ball to the center when the tablet is flat. Participants have to tilt the tablet to rotate the ball around the center. Each participant controls only one axis, either x or y, by moving the index finger up and down. The lever on the x-axis is longer in order to compensate the different edge lengths of the tablet. The tablet is limited to two degrees of freedom (DOF) and prevents any rotation (see Figure 2). The task for the participants is to rotate the ball around the center while following the circular target trajectory as precisely as possible. The ball's circular movement can be realized when both axes of the tablet are tilted in a certain pattern and with a certain amplitude of frequency. Optimal performance is achievable with synchronization of the finger movements with a 90° phase difference (see Video 1 in the Supplementary Material).

Figure 2 shows the side view of the experimental setup. Participants tilt the tablet up and down through the levers that are attached to the casing. The tablet is supported by a universal joint that allows rotations on the x- and y- axis (roll and pitch), but prevents rotations around the z-axis (yaw). To avoid hand movements other than up-and-down movements of the index finger, participants were asked to hold the handle that was fixed to a wooden frame which is shown in Figure 2. Participants can comfortably rest their elbows on the layer 2 of the wooden frame.

**Figure 3:**
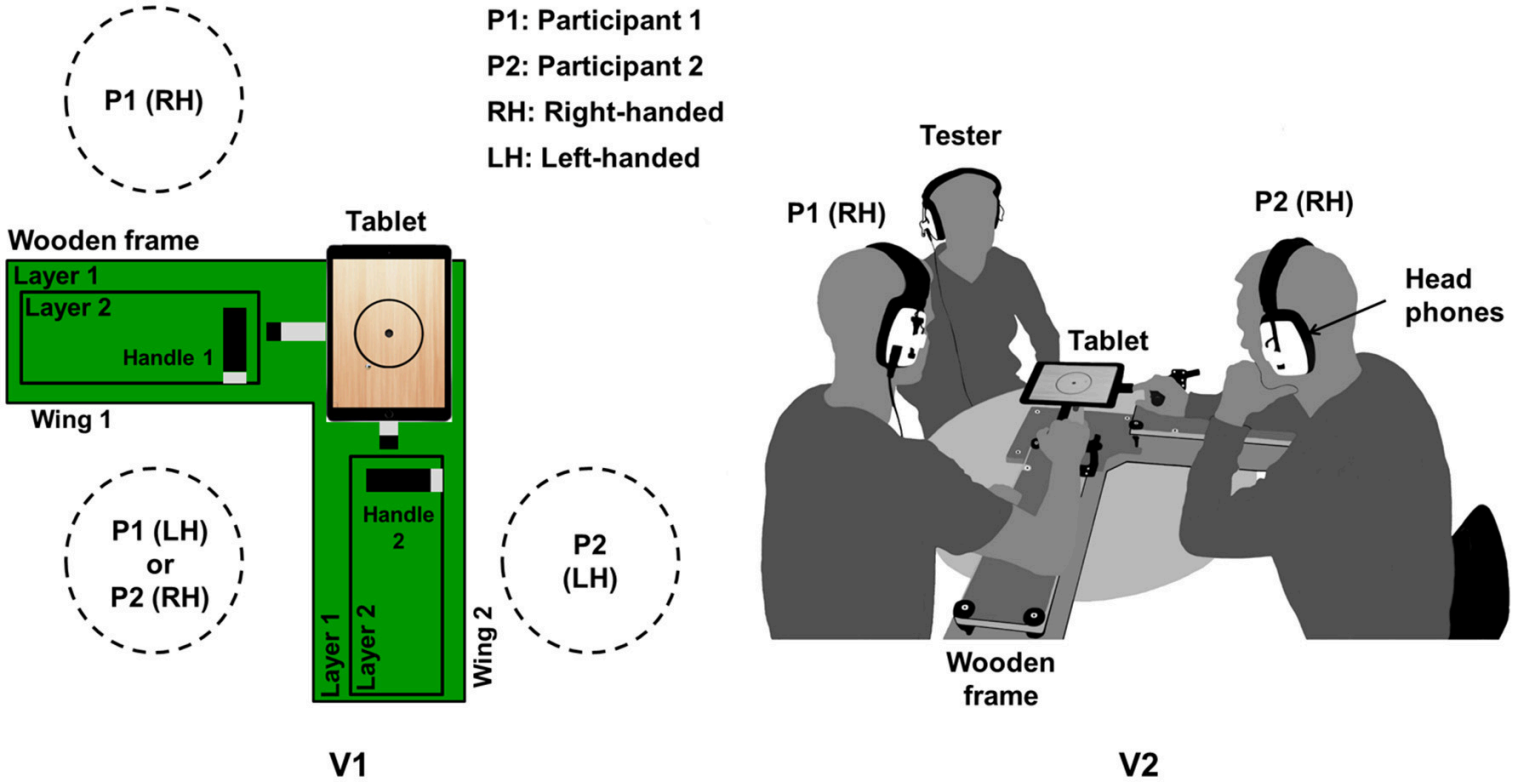
Illustrations of **(V1)** top view of the task apparatus and the seat plan for right-handed (RH) and left-handed (LH) participants (P1 and P2), as well as **(V2)** positions of two right-handed participants and a tester during the experiment.

Figure 3 shows the top view of the tetherball paradigm including the wooden frame. Participants sit to control the tablet by using their dominant hand. Right-handed (RH) participants sit on the left of a wooden frame's wing and left-handed (LH) participants sit on the right of a wing. The participants stay on their seats during the whole task and do not swap position. The handles can be adjusted to the dominant hand and to the hand size of each participant. Participants can see the screen from nearly the same distance, which establishes the same condition for visual feedback. They wear headphones for auditory feedback. The audio output of the tablet is connected to an audio splitter, and the participants hear the same sound at the same time. They hear their own and their partner's auditory feedback.

**Figure 4:**
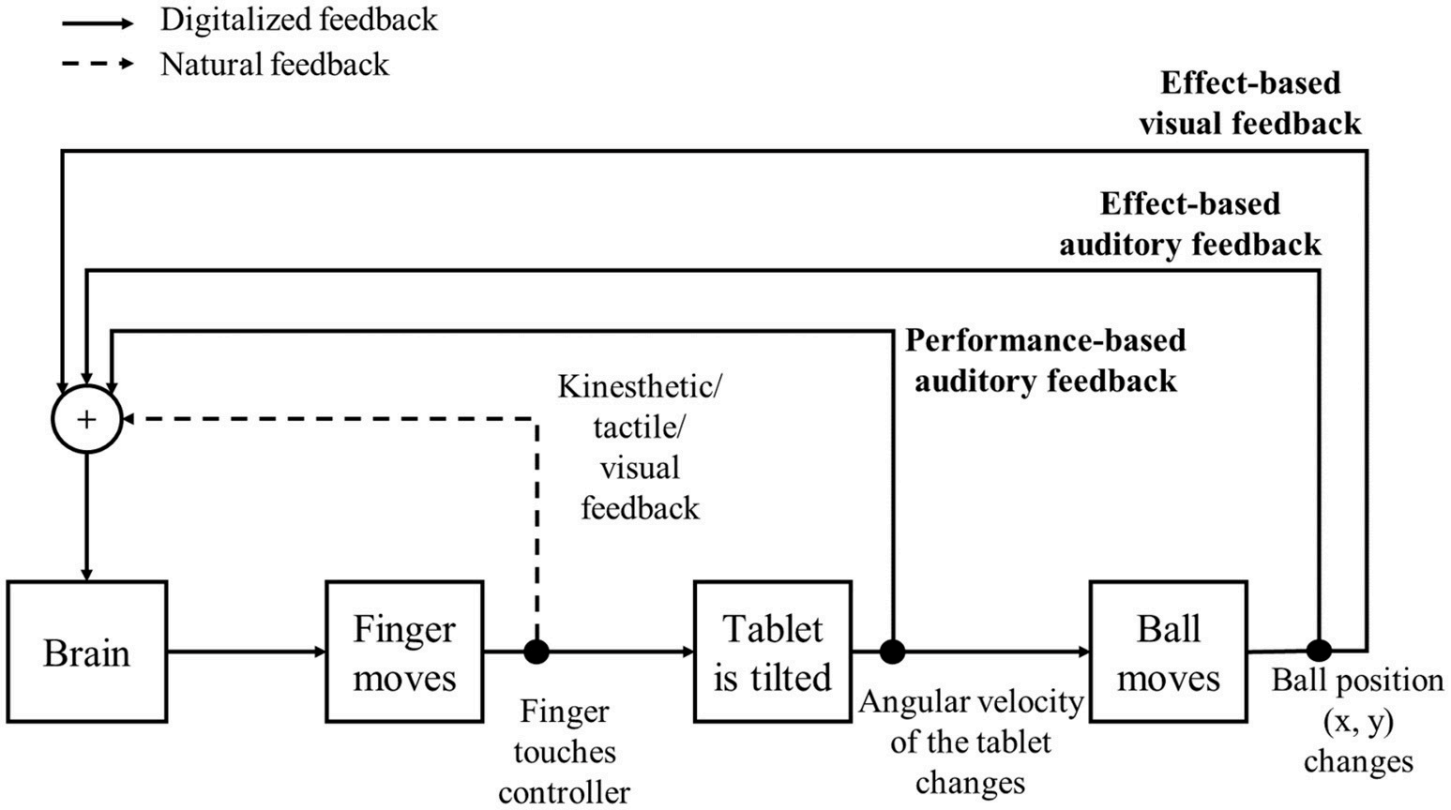
The feedback modeling in perceptual information flows based on types of feedback.

Figure 4 shows the perceptual information flows including visual, auditory, tactile, and kinesthetic information. Effect-based visual (VF), EAF, and PAF are digitally treated as experimental variables, whereas the kinesthetic, tactile, and visual feedback of finger movements are independent variables in this paradigm. The ball moving through the scene constitutes VF. Effect-based auditory feedback is driven by the position of the ball, which is congruent to VF. For EAF, “synthesized violin” is used to create continuous string instrument sound so that it is appropriate to sonify the ball's continuous movement pattern. Two distinguishable violin sounds can also be converted from two spatial parameters, the x- and y-position. The sound is, furthermore, familiar to human ears because it can mimic the human voice in terms of range of spectrum and vibration, wherein participants can hear the sound for a relatively long time. To be specific, EAF is represented by pitch and amplitude of the sound. The pitch of the sound corresponds to the x- and y-position, whereas the amplitude depends on the ball's velocity. Depending on the ball position on the tablet's screen, the base audio frequency is modified from 250 to 427 Hz along the x axis and from 600 to 835 Hz along the y axis.

Performance-based auditory feedback represents the angular velocity of the tablet measured by the built-in gyroscope. When the tablet is tilted, the resulting angular velocity affects PAF as additional auditory feedback about the participant's actions—convergent with their kinesthetic finger perception. The sound of PAF is created by a noise generator with a band-pass filter, which is a “broom sweeping sound.” We decided to use this sound because it is suitable to express accelerating up-and-down finger movements of participants. Spectra of both tilt sounds are easily distinguished because they were located within different frequency bands. This timbre is closely related to natural sounds so that participants can hear it comfortably during the task. The PAF sound also allows the participant to clearly distinguish it from EAF in the CAF condition. Higher velocity of finger movements generates a higher amplitude and frequency of the PAF sound. Depending on the centrifugal force from accelerometer data, the base frequencies (*f*_*b*_) are 700–1,700 Hz for lever 1's tilt and 100–1,100 Hz for lever 2's tilt, respectively. We obtained the sound from the white noise after using the band-pass filter (cutoff frequency: *f*_*b*_ ±25 Hz). Together, the auditory feedback generates rhythmical sounds corresponding to the periodic finger movements with altering velocities and short phases of silence at the turning points. Besides these two types of augmented auditory feedback (PAF, EAF), participants also had natural kinesthetic, tactile and visual feedback to solve the experimental task. A sample video of the tetherball paradigm with additional auditory feedbacks is provided in Video 1 (in the Supplementary Material).

### Procedure

**Figure 5:**
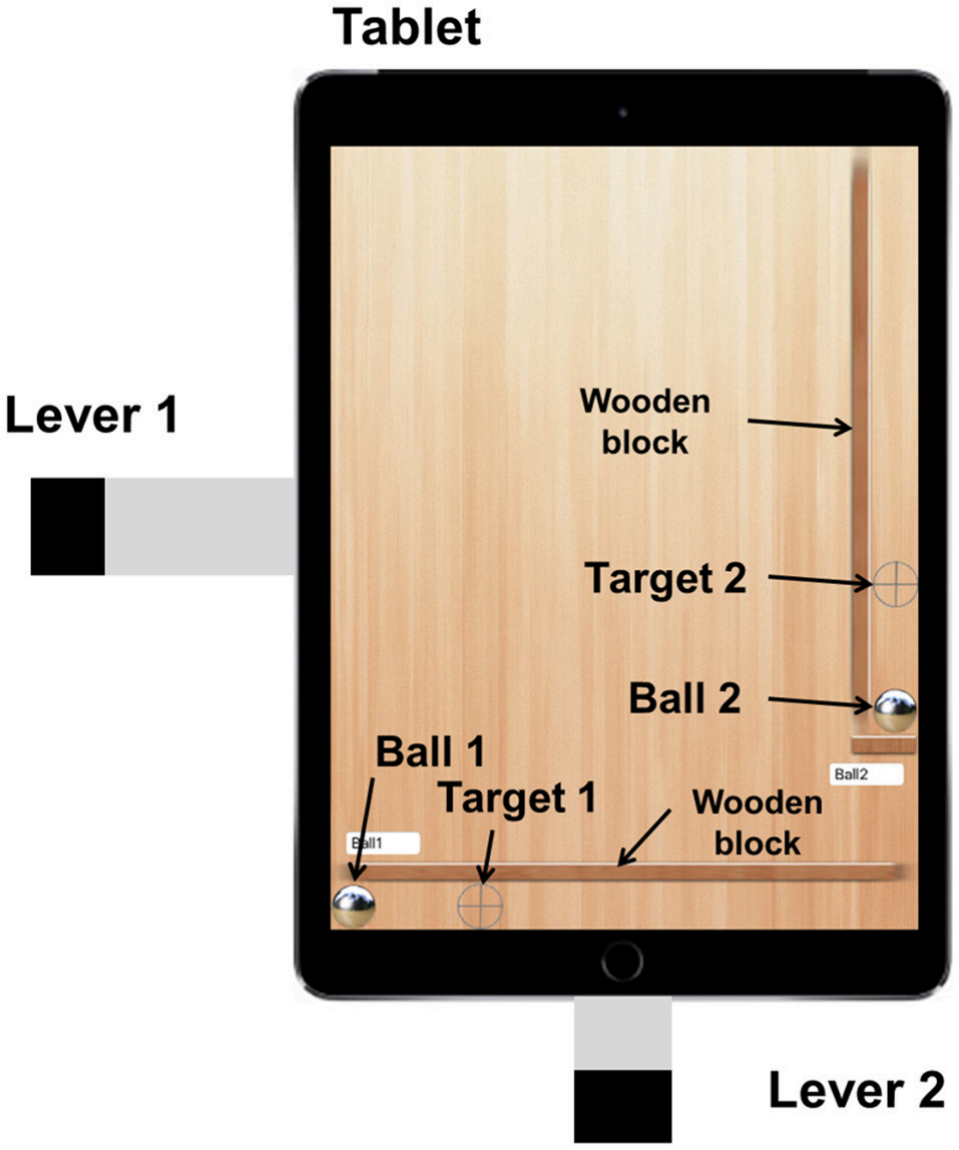
Top view of pre-test for measuring initial performance.

Before the experiment, participants were asked to complete a questionnaire regarding their personal backgrounds including previous experiences in music and sports settings. Two pre-tests were administered to confirm that the participants have a normal range of eyesight and hearing abilities, which were tested with the Landolt rings chart (Jochen Meyer–Hilberg) and HTTS audio test (SAX GmbH). The third pre-test was carried out to classify participants depending on their ability to handle the ball on the screen, which might decide their performance in the pre-test shown in Figure 5. Participants have to keep the balls on randomly moving targets. Each participant handled a separated ball moving along the corresponding axis.

The participants performed the visuo-motor pre-test for 2 minutes. For each participant, the mean absolute error (the distance between the target and ball position) was measured during the last 30 s. The performance of this task and participants' gender was used for parallelization between groups: The first four pairs were randomly assigned to four groups. The visual group (VFG) received VF without auditory feedback as a reference condition. The EAF group (EAFG), the PAF group (PAFG), and the CAF group (CAFG) additionally received EAF, PAF, and CAF, respectively. All groups also received natural kinesthetic, tactile, and visual feedback which was not modified in the experiment. Group assignment of all other pairs considered their mean error in the pre-test. Thereby, it was possible to compose four groups with nearly the same level and without statistically different visuo-motor pre-test performances [VFG: 75 ± 23 px, EAFG: 72 ± 29 px, PAFG: 70 ± 20 px, CAFG: 78 ± 16 px; *F*_(3, 36)_ = 0.24, *p* = 0.872, η_p_^2^ = 0.02].

Couples of participants performed 15 trials of 1 min each. After every five trials, a 2-min break was administered, resulting in three sets. During the trial, participants abstained from talking and discussing about possible strategies, so that they could focus on the task. Participants were also instructed to initiate the revolving of the ball in clockwise direction (CW). After the experiment, the participants were asked to answer the second questionnaire that assessed subjective ratings of participants' experience in terms of interpersonal coordination at solving the task. The questionnaire consisted of four questions subjectively evaluating their personal, their partners', and the joint performance during the experiment.

### Data analysis

The tablet recorded the path of the ball (from screen) and the angular velocity (from gyroscope) at the sampling rate of 60 samples per second. For statistical analysis, absolute tracking errors as well as mean peak values from the cross correlations were submitted to three-way analyses of variance with a between-subject factor Group (VFG vs. EAFG, VFG vs. PAFG, VFG vs. CAFG) and the within-subject factors Set (I–III) and five Trials in each set. The sphericity assumption was tested with the Mauchley's test, and in case of significance, ANOVAs were adjusted according to the Huynh–Feldt procedure. Levene's test was applied to analyze homogeneity of variances. *Post-hoc* comparisons were performed with Tukey's *post-hoc* tests. Subjective ratings of interpersonal coordination were compared across groups with Mann–Whitney-*U*-Tests and within groups with a Wilcoxon test. The overall significance level was set to 5%.

## Results

Sport-, music-, and computer-game-expertise, as well as pre-test performance were taken into account because they could influence performance in the tetherball paradigm. Comparing these variables of groups with those of the VFG, we found no significant differences in these variables except for sport expertise between the VFG and PAFG [*F*_(1, 16)_ = 6.38, *p* = 0.022, η_p_^2^ = 0.29]. Therefore, we considered sport specific expertise as a possible covariate in the subsequent analyses.

**Figure 6:**
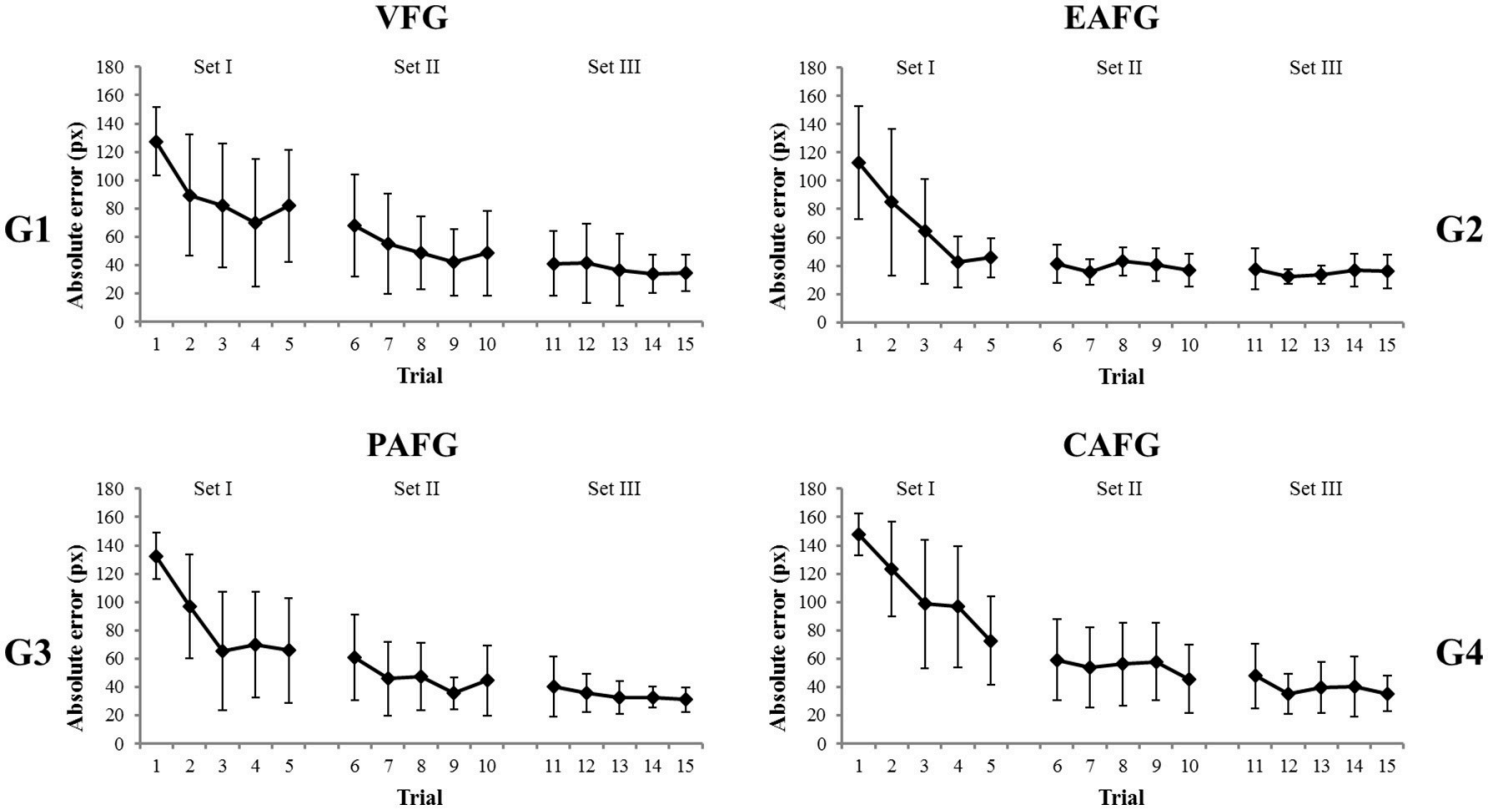
Across subject means and standard deviations of the absolute error over the trials in four groups, **(G1)** the VFG, **(G2)** the EAFG, **(G3)** the PAFG, and **(G4)** the CAFG. Illustrated are between-subject means and standard deviations. The first 8.3 s (500 samples) in every 1-min trial was eliminated.

The performance was measured by the absolute error between the radius of the target circular trajectory and the ball's trajectory. An average value of the absolute error during a 1-min trial was calculated; however, data of the first 8.3 s (500 samples at 60 Hz) in every 1-min trial were omitted because the circling ball's movement had to be initiated. With the average absolute error, we calculated across subject means and standard deviations for each trial and in each group (Figure 6).

The mean absolute errors of four groups are shown in Figure 6. Comparing the results of VFG and EAFG across trials, the absolute error decreased significantly from Set I to Set II and Set III as confirmed by the significant effect set [*F*_(2, 30)_ = 3.95, *p* = 0.043, $\eta_{\text{p}}^{2}$ = 0.21] and significant differences between Set I to Set II (*p* < 0.001) as well as Set I to Set III (*p* < 0.001) in the *post-hoc* test. Furthermore, within each set, the error decreased from Trial 1 to 5 [*F*_(4, 60)_ = 4.58, *p* = 0.005, η_p_^2^ = 0.23]. A *post-hoc* comparison confirmed significant differences between Trial 1 and all the other trials (each *p* < 0.001) and between Trial 2–4 and 5 (both *p* < 0.01). For the error reduction across trials, sport specific expertise was the significant covariate [*F*_(4, 60)_ = 3.84, *p* = 0.013, η_p_^2^ = 0.20].

The error reduction differed between groups as confirmed by the three-way interaction Set^*^Trial^*^Group [*F*_(8, 120)_ = 2.63, *p* = 0.030, η_p_^2^ = 0.15]. The participants in EAFG predominantly increased their performance within the first four trials and then reached a stable plateau. Accordingly, a *post-hoc* test showed significant differences from the first three trials to the last trial of the task (at least *p* < 0.05), but no significant differences from Trial 4 onwards (all *p* > 0.05). The error of the VFG reached a plateau at the same level as that of the EAFG but at a later trial. Thus, the *post-hoc* test confirmed significant differences between the first six trials (Trial 1–6) and the last three trials (Trial 13–15) in the task (at least *p* < 0.05). Levene's test revealed that variances differed significantly between groups in Trials 4–8 and Trial 12 (at least *p* < 0.05).

In contrast to the EAF, the PAFG did not show a significant difference in performance, compared to the VFG. A comparison of the absolute error with the VFG neither resulted in significant group differences nor interactions. Across groups, however, became significant in terms of the main effects, set [*F*_(2, 32)_ = 56.66, *p* < 0.001, η_p_^2^ = 0.78] and trial [*F*_(4, 64)_ = 40.81, *p* < 0.001, η_p_^2^ = 0.72] as well as their interaction [*F*_(4, 64)_ = 10.19, *p* < 0.001, η_p_^2^ = 0.39]. A *post-hoc* test to the latter interaction confirmed significant differences from Trial 1–2 to Trial 3–5 in Set I (at least *p* < 0.05), significant differences from Trial 6 to Trial 9–10 in Set II (at least *p* < 0.05), and no significant difference between the trials in Set III (all *p* > 0.05). This indicated that the performance increased predominantly in Set I and reached a plateau in Set III. The Levene's test was not significant in any the other trials.

An ANOVA for VFG and CAFG yielded the same overall effects as the other ANOVAs [Set: *F*_(2, 32)_ = 67.26, *p* < 0.001, η_p_^2^ = 0.81; Trial: *F*_(4, 64)_ = 35.76, *p* < 0.001, η_p_^2^ = 0.69] as well as a significant interaction in Set^*^Trial [*F*_(4, 64)_ = 10.56, *p* < 0.001, η_p_^2^ = 0.40]. Furthermore, the CAF had a significant effect on the progress of error reduction, which is confirmed by significant interactions in Trial^*^Group [*F*_(4, 64)_ = 3.70, *p* = 0.021, η_p_^2^ = 0.19]. Here, a *post-hoc* test confirmed that the CAF allowed the participants to further increase their performance from the second last to the last trial (*p* = 0.02). This was not the case in VFG (*p* > 0.05). Furthermore, the significant three-way interaction in Set^*^Trial^*^Group [*F*_(8, 128)_ = 2.45, *p* = 0.031, η_p_^2^ = 0.13] indicated that the error reduction progressed differently between groups. In CAFG, the performance reached a plateau earlier than in VFG. According to Tukey's *post-hoc* test, the first five trials (Trial 1–5) in CAFG differed significantly from the last trial (all *p* < 0.001). In VFG, the first six trials (Trial 1–6) differed significantly from the last trial (all *p* < 0.001). Levene's test was not significant in any of the trials.

**Figure 7:**
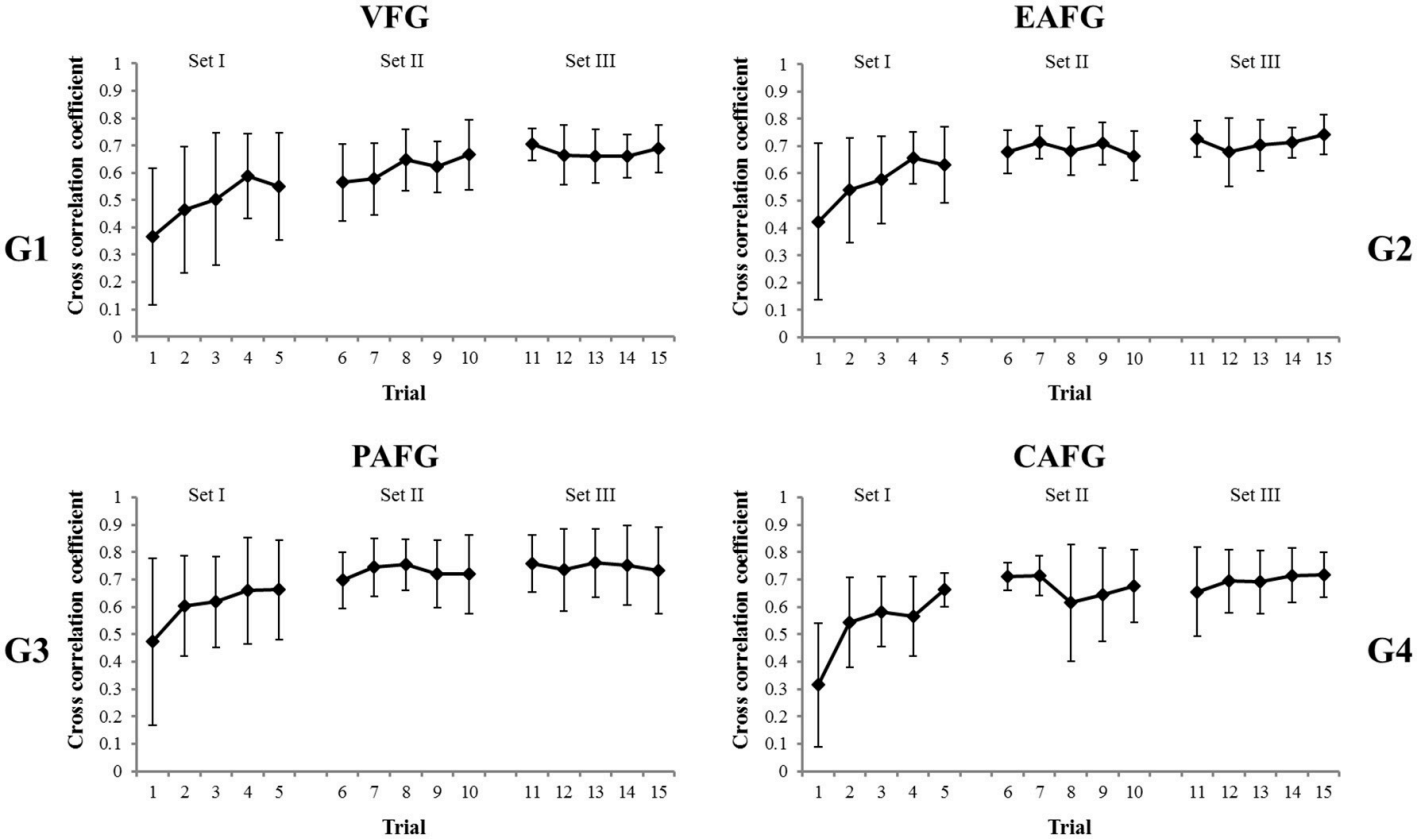
Across subject means and standard deviations about the cross correlation coefficient of a pair of participants over the trials in four groups, **(G1)** the VFG, **(G2)** the EAFG, **(G3)** the PAFG, and **(G4)** the CAFG. To take maximal performance of pairs, the maximal coefficient in each trial were chosen with lags equivalent about 90° phase delay. The first 8.3 s (500 samples) in every 1-min trial was eliminated.

Regarding the level of temporal synchronization, we calculated the cross correlation between the angular velocities of a pair of participants' up-and-down finger movements, which is applied to all other pairs (Figure 7). Cross-correlation was calculated with 1,000 samples, and then this was divided into three periods in each 1-min trial (3,610 samples, 60.2 s). A calculation of the cross-correlation resulted in coefficients along with lags (*n* = ±50). Parts of coefficients were considered, especially when the lags were between 8 and 15 samples. These values were empirically determined as a standard, regarding quarter-phase synchronization. To decide the optimal lag values, we selected the best 12 pairs (three pairs per group) who achieved the lowest average error of ball trajectory during the last five trials (Trial 11–15). We measured an average time difference equivalent to a 90° phase difference between a pair of participants' angular velocities. The time difference was 194.2 ± 75.5 ms corresponding to 11.65 (±4.53) samples of the lag. According the calculations, the highest coefficient was extracted between 133.3 ms (*n* = 8) to 250.0 ms (*n* = 15). Then, we had three coefficients (*n* = 500–1,500, 1,500–2,500, and 2,500–3,500) in every 1-min trial (*n* = 3,610) for every pair. The largest coefficient in a 1-min trial was regarded as a representative value for the trial. This allowed us to record the best performance of pairs in each trial. This is because we can avoid the average effect of participant's mistakes. The first 8.3 s (*n* = 500) were eliminated, because it was before the ball was released. From these three sections, the maximum coefficient for a single trial was selected. According to across subject means and standard deviations of the coefficients shown in Figure 7, the correlations improved over time. This was statistically confirmed by significance of the factor “set” in the ANOVAs which analyzed the data of the VFG and audio-visual groups [VFG & EAFG: *F*_(2, 32)_ = 26.81, *p* < 0.001, η_p_^2^ = 0.63; VFG & PAFG: *F*_(2, 32)_ = 21.17, *p* < 0.001, η_p_^2^ = 0.57; VFG & CAFG: *F*_(2, 32)_ = 26.82, *p* < 0.001, η_p_^2^ = 0.63] as well as the significant effects of trial [VFG & EAFG: *F*_(4, 64)_ = 5.49, *p* = 0.003, η_p_^2^ = 0.26; VFG & PAFG: *F*_(4, 64)_ = 5.48, *p* < 0.001, _*p*_^2^ = 0.26; VFG & CAFG: *F*_(4, 64)_ = 8.68, *p* < 0.001, η_p_^2^ = 0.35]. The improvement of cross-correlation changed over time as shown by the significant interactions Set^*^Trial in these groups [VFG & EAFG: *F*_(8, 128)_ = 5.25, *p* < 0.001, η_p_^2^ = 0.25; VFG & PAFG: *F*_(8, 128)_ = 4.68, *p* < 0.001, η_p_^2^ = 0.23; VFG & CAFG: *F*_(8, 128)_ = 6.20, *p* < 0.001, η_p_^2^ = 0.28]. Furthermore, cross correlations increased significantly faster with CAF than without auditory feedback (VF). This is confirmed by the significance of the three-way interaction Set^*^Trial^*^Group [*F*_(8, 128)_ = 2.53, *p* = 0.014, η_p_^2^ = 0.14]. Accordingly, a Tukey's *post-hoc* test results in significant differences between the first three trials and the last trial within VFG (each p at least <0.05), whereas in CAFG only the first two trials differed significantly from the last (each p<0.05).

**Figure 8:**
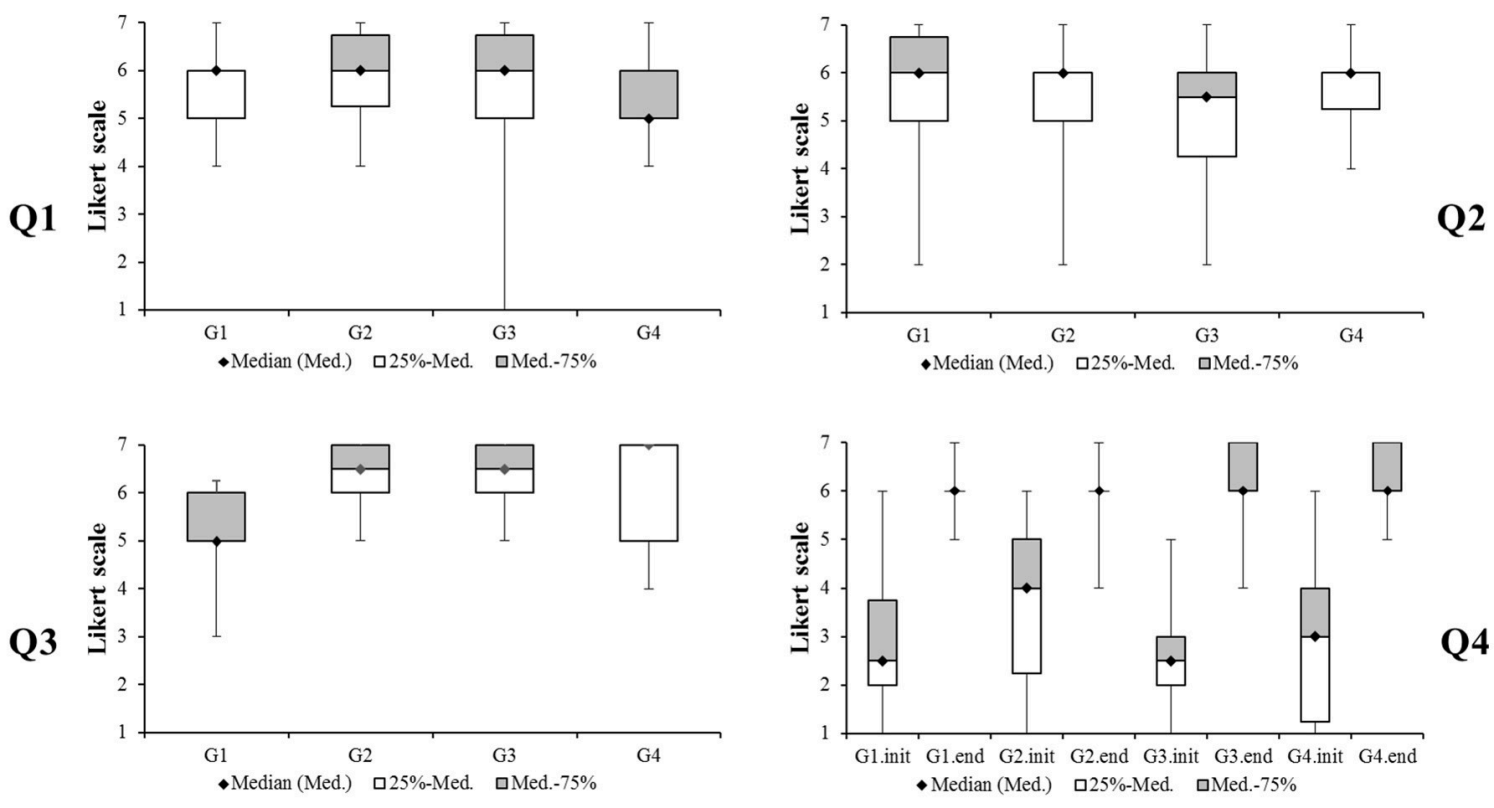
Box and whisker plots of subjective ratings when participants were asked about how much they felt **(Q1)** that their own action helped their partners, **(Q2)** that their partners' action helped their own action, **(Q3)** how pleasant was their experience during the collaboration, and **(Q4)** how effectively they felt that they handled the apparatus together at the initial time and at the end.

Results of the questionnaire are shown in box and whisker plots in Figure 8. Participants were asked to choose an integer between 1 (not at all) and 7 (very much), when answering the first question “How much did you feel your movement helps the collaborator's performance?” All participants answered without significant difference between VFG and audio-visual groups (EAFG, PAFG, CAFG) according to Mann–Whitney *U*-tests (VFG vs. EAFG: U = 150.0, *p* = 0.719; VFG vs. PAFG: *U* = 161.0, *p* = 0.988; VFG vs. CAFG: *U* = 120.0, *p* = 0.192). Participants normally scored between 4 and 7. Medians of all groups were between 5 and 6. The second question “How much did you feel the collaborator's movement helps your performance?” also resulted in no significant differences (VFG vs. EAFG: *U* = 159.5, *p* = 0.938; VFG vs. PAFG: *U* = 136.0, *p* = 0.424; VFG vs. CAFG: *U* = 159.0, *p* = 0.938). However, in the third question “How did you experience the collaboration with your partner?” participants were asked to mark from 1 (unpleasant) to 7 (very pleasant). The ratings audio-visual groups showed significant differences to the VF group (VFG vs. EAFG: *U* = 66.0, *p* = 0.002; VFG vs. PAFG: *U* = 60.0, *p* = 0.001; VFG vs. CAFG: *U* = 90.5, *p* = 0.022). The fourth question “How effectively did you feel that you managed to do the task?” was asked to be marked from 1 (not effectively at all) to 7 (very effectively) for their feeling at the beginning and at the end of experiment. Results of rating by EAFG showed a higher median value at the initial time than VFG, and there was a tendency of difference between VFG and EAFG (*U* = 103.0, *p* = 0.064). However, neither this nor other differences between groups were significant (VFG vs. PAFG: *U* = 151.0, *p* = 0.743; VFG vs. CAFG: *U* = 156.0, *p* = 0.864). In comparison to the beginning of the experiment, participants felt that they managed the task more effectively at the end as shown by a significant effect in the Wilcoxon-Test (*z* = −7.28, *p* < 0.001). Noteworthy, the progress from the initial time to the end, calculated as pre-post difference, was not significantly different between groups (VFG vs. EAFG: *U* = 112, *p* = 0.118; VFG vs. PAFG: *U* = 156, *p* = 0.864; VFG vs. CAFG: *U* = 128, *p* = 0.293).

## Discussion

In the tetherball paradigm, participant pairs were asked to tilt the tablet together for the task. We compared three different audio-visual conditions with the visual condition in terms of error reduction, cross correlation and subjective ratings in a self-report questionnaire. Results demonstrate that error reduction was faster with EAF and CAF than the visual condition; however, no statistical difference was observed with PAF. This confirms H1(a) and H1(c), but not H1(b). Regarding H2, only H2(c) is supported by our results, because only CAF showed a significant effect on the cross correlation between participants compared to the visual condition. In terms of H3, participants hardly perceived that their actions affected their partner's action and vice versa. Nonetheless, participants with auditory feedback felt more pleased in the collaborative task than those without auditory feedback. Across groups, participants felt progress in collaboration; however, differences between visual and audio-visual groups were not significant. Therefore, H3 can be partially confirmed in terms of pleasant feeling during the task by the present study.

The task required the participants to predict their partner's actions as well as the combined effect of their joint actions. Our results suggest that real-time audio-visual feedback improved performance. According to Stein and Stanford (2008), perception can be usually enhanced if visual and auditory information are integrated within multisensory areas of the central nervous system (CNS). This might enhance participants' understanding of their own and their partner's actions as well as joint actions, which positively affects interpersonal coordination. In addition, previously published literature (Schmidt and Richardson, 2008; Keller et al., 2014; Lang et al., 2016; Loehr and Vesper, 2016) highlights the significance of rhythmical movement components in interpersonal coordination. Additionally, there is evidence that the rhythmic component during interpersonal coordination reduces practice effort and errors (Lang et al., 2016; Loehr and Vesper, 2016). When rhythmical information of the movement is shared between two or more individuals by visual or auditory cues, usually spatiotemporal entrainment is supported by the same dynamical principles of the movement (Knoblich et al., 2011; Phillips-Silver and Keller, 2012). According to Schmidt and Richardson (2008), moreover, additional perceptual information can increase the level of action coupling, possibly enabling co-actors to align their actions. In our setting, EAF contained non-rhythmical sound; however, it provided a temporally structured melody. This sound could have aided the participants to predict the ball dynamics, to estimate the achieved precision, and to adapt further actions. Furthermore, after reaching the plateau level of performance until the end of the task, the absolute error in EAFG showed significantly lower standard deviations than VFG. This might indicate that participants maintained interpersonal coordination more consistently after establishing a task-specific audio-visual-motor network in the brain.

However, PAF alone caused no significant effect on error reduction and cross correlation. This result indicates different effects of various types of auditory information on interpersonal coordination. A plausible explanation is the integration of auditory information with perceptual information of other modalities in terms of multisensory integration. For example, Allerdissen et al. (2017) reported that fencing experts showed nearly the same pattern of results in both audio-visual and visual conditions. A similar suggestion had been made by Demos et al. (2012): The authors showed that the level of coordination can be enhanced by audio-visual information, but can be reduced by non-task-related auditory stimuli like music. In our setting, the characteristics of the chosen sounds may also have influenced the results. The EAF sound (“synthesized violin”) was a more dominant auditory cue than the PAF sound because it was a continuous sound with high pitch and bright timbre. If we used other sounds similar to the “synthesized violin” of the EAF condition, PAF could have enhanced interpersonal coordination. Of course, not only the chosen timbre of the sound can change the way it is perceived, but also the determined level of volume as well as masking effects between both sounds. We nevertheless, tried to find well balanced compositions where both sounds were equally perceivable well. Finally, PAF had neither positive nor negative effects on interpersonal performance compared to the visual group (VFG) in our study.

As CAF, we used PAF and EAF together to investigate the effect when more types of additional auditory information were applied additionally to VF, expecting enhanced performance without the need of conscious attention (see Effenberg et al., 2016). Interpersonal coordination was significantly affected by CAF in terms of enhanced joint performance and temporal synchronization. The effect on joint performance can be explained by the presence of EAF because PAF did not show an effect. The effect on temporal synchronization, nevertheless, might be supported by the combination of EAF and PAF. Although PAF alone does not affect interpersonal coordination, it seems there is a synergy between PAF and EAF.

Our results suggest that additional auditory feedback can make collaboration easier and more pleasant. Effect-based auditory feedback can increase motivation for the task because participants in audio-visual groups reported that they felt more pleased during interpersonal coordination. Most interestingly, PAF also resulted in a similar pleasing effect. Demos et al. (2012), for instance, reported that music irrelevant to vision and movement made participants feel connected with their partners. This might suggest that the pleasant feelings are rather related to the auditory task component than to task performance. For future research, it might be interesting to investigate whether participants feel pleased during the task with non-task-related auditory feedback (rhythmical, non-rhythmical). This would be in line with a study of Phillips-Silver and Keller (2012) on affective entrainment when the authors investigated the relations between the task-relatedness of a sound and the pleasantness of the participants' feelings in the synchronization with others.

In future, auditory feedback might be applied to facilitate interactions between humans and machines. Humans possess an ecological acoustic-motion mapping background based on every-day experiences (Carello et al., 2005): For example, when driving a car, the engine sound correlates with its speed. Such movement sounds like a washing machine, a vacuum cleaner, and a printer might be regarded as performance-based feedback. Other examples suggest that many humans are also experienced with ecological or artificial effect-based auditory feedback: A modern car provides the driver with artificial auditory feedback about the distance to objects during parking, and a radar sonifies the distance and velocity of approaching objects. In these scenarios, machines mediate information via audition to humans. As the present study represents a first step in the case of human-human interaction, future studies might investigate which sounds support human-machine interactions best. The adequate choice of an appropriate auditory coding of physical performance and events is important. As already stated, out results suggest that certain kinds of human-human interaction benefit from effect-based auditory information, at least, if the common goal is already known. In the case of humanoid human-robot interaction scenarios it might not be possible to predict joint effects as long as referenced actions have not been experienced before. For such underdetermined, novel interaction scenarios it might be useful to apply a performance-based acoustics in a first step. Although we did not find benefits of exclusively performance-based auditory information in our study, humanoid robot-human interactive settings might benefit from additional performance-based kinematic real-time acoustics: With reference to Schmitz et al. (2013), auditory information about humanoid robotic movements might be suitable to address biological motion perception mechanisms in the human brain, if configured adequately. Biological motion perception mechanisms are usually not addressed by artificial agents with non-human motions.

## Conclusion

Additional artificial auditory information can be synthesized in many different ways for interpersonal coordination. In this study, we referred to the feedback research in the motor domain with a basic reference to the both categories of “knowledge of performance” (KP) and “knowledge of result” (KR), well-established in motor learning research. In future, it might be interesting to investigate relationships between sounds and movements in various situations with more difficult levels of joint tasks with long-term period (e.g., shape-changing trajectory). An important aspect of further research is how motor learning and the emergence of interpersonal coordination are related to each other. Undoubtedly both are referring closely to the perception of kinematics—mainly dedicated to human movements or to the referenced object's movements (e.g., a sofa, a tetherball). To support the perception of kinematics might be a key issue on many places in future—related to the support of individual behavior as well as of interpersonal coordination. Nevertheless, it is a challenging approach—related to motor learning and to interpersonal coordination.

## Author contributions

T-HH together with AE and GS developed the paradigm and the experimental design. T-HH realized the software development supported by HB. T-HH organized the database and wrote the technical parts of the paper. AE and SG wrote main parts of the behavioral sections of the paper. AE and GS supervised the data collection. KK and LB performed the experiment. Statistical analysis and major parts of the results were realized by GS, supported by T-HH. The software development of the visuo-motor pre-test as well as the development of the mechanical parts of the apparatus were realized by MS and AM. All authors critically revised the manuscript.

### Conflict of interest statement

The authors declare that the research was conducted in the absence of any commercial or financial relationships that could be construed as a potential conflict of interest.
