# Supplementary material for: Effect- and Performance-Based Auditory Feedback on Interpersonal Coordination
Source: Front Psychol. 2018 Mar 29;9:404. doi: 10.3389/fpsyg.2018.00404 (PMC5885253; doi:10.3389/fpsyg.2018.00404)
Supplement: Supplementary file 1 [file Presentation1.pptx]

## Slide 1
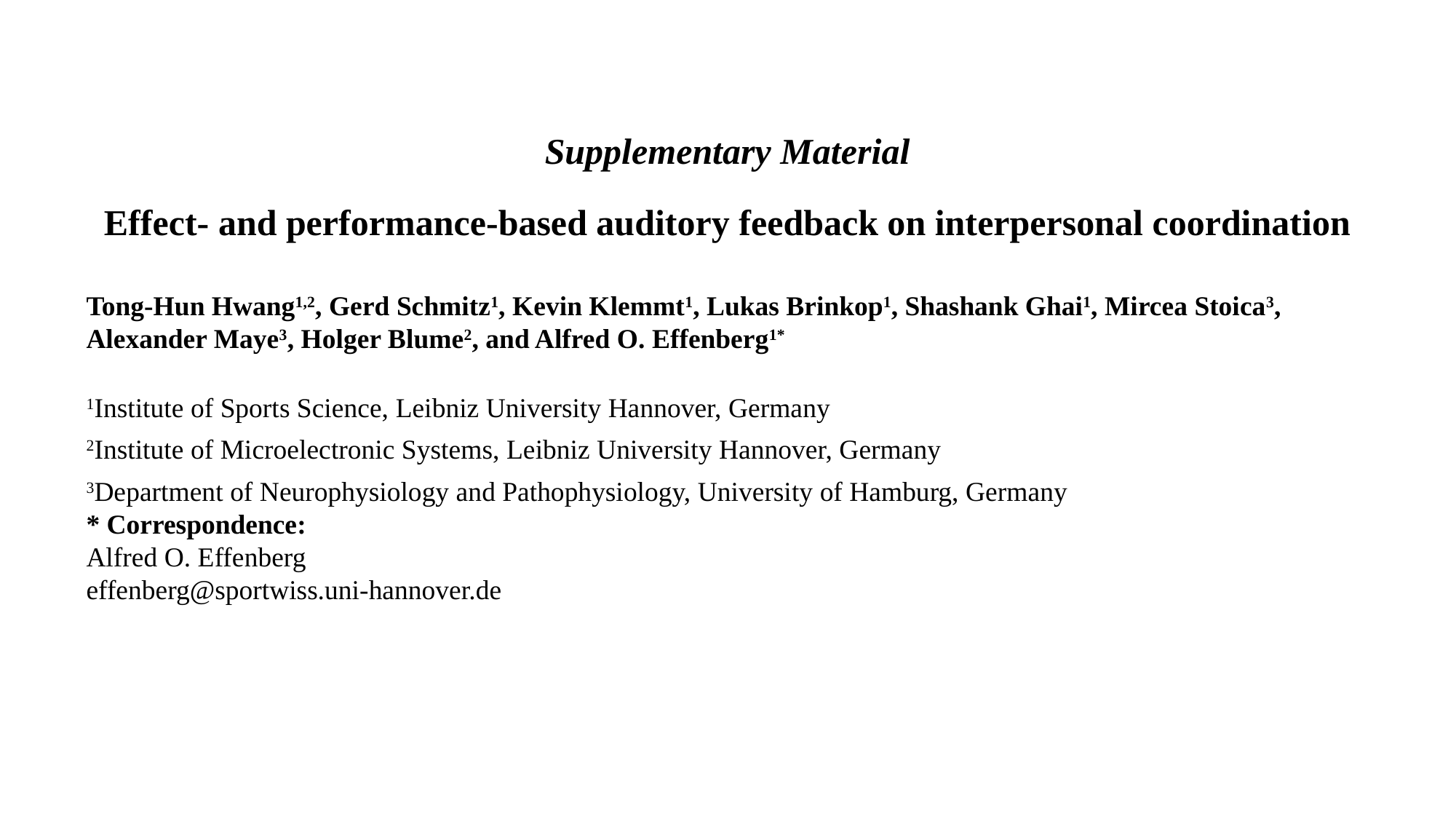

Supplementary Material
Effect- and performance-based auditory feedback on interpersonal coordination
Tong-Hun Hwang1,2, Gerd Schmitz1, Kevin Klemmt1, Lukas Brinkop1, Shashank Ghai1, Mircea Stoica3, Alexander Maye3, Holger Blume2, and Alfred O. Effenberg1*
1Institute of Sports Science, Leibniz University Hannover, Germany
2Institute of Microelectronic Systems, Leibniz University Hannover, Germany
3Department of Neurophysiology and Pathophysiology, University of Hamburg, Germany
* Correspondence:
Alfred O. Effenberg
effenberg@sportwiss.uni-hannover.de

## Slide 2
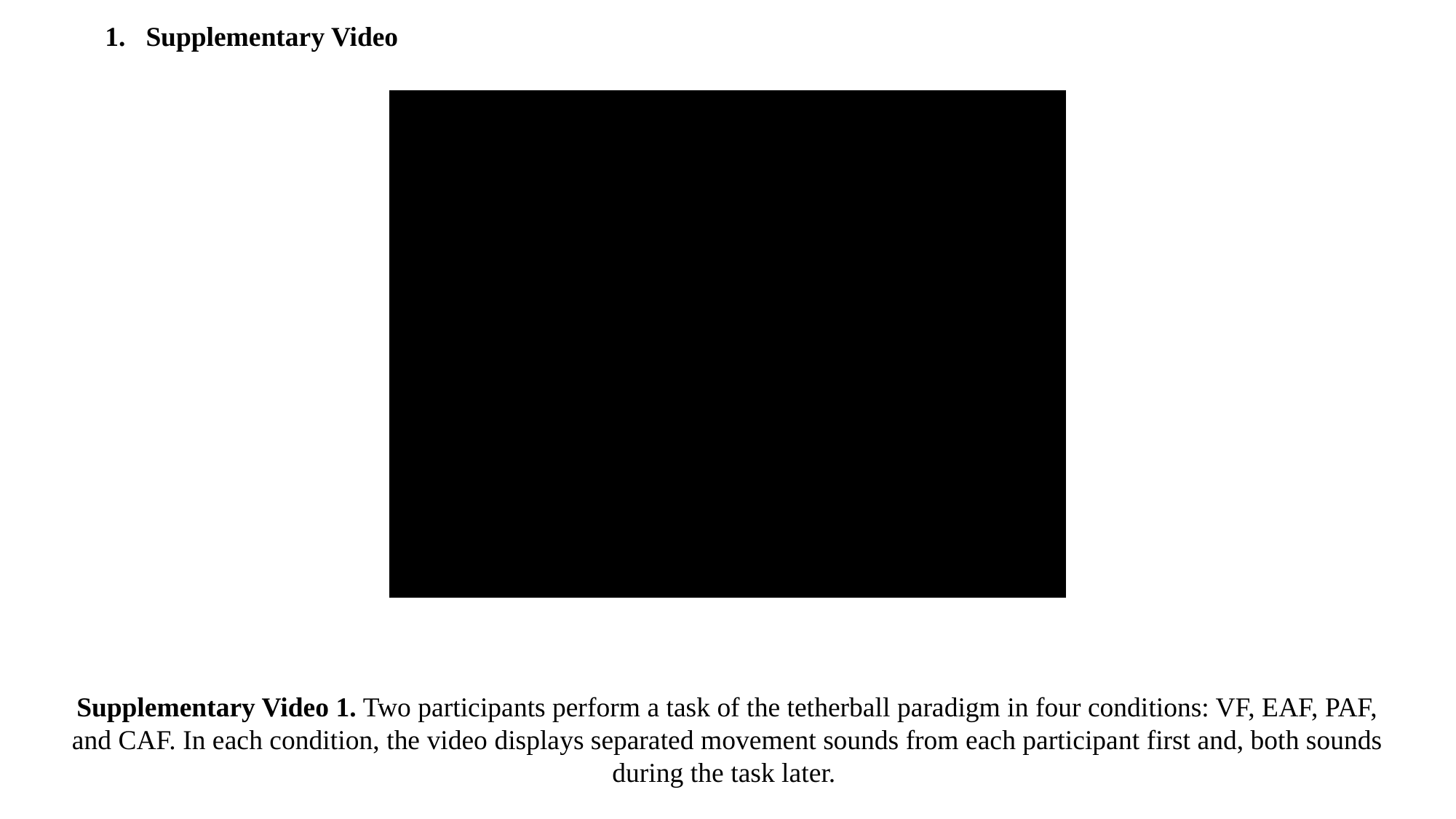

Supplementary Video
Supplementary Video 1. Two participants perform a task of the tetherball paradigm in four conditions: VF, EAF, PAF, and CAF. In each condition, the video displays separated movement sounds from each participant first and, both sounds during the task later.
